# Differences in Immune Responses in Individuals of Indian and European Origin: Relevance for the COVID-19 Pandemic

**DOI:** 10.1128/spectrum.00231-23

**Published:** 2023-02-13

**Authors:** Büsra Geckin, Martijn Zoodsma, Gizem Kilic, Priya A. Debisarun, Srabanti Rakshit, Vasista Adiga, Asma Ahmed, Chaitra Parthiban, Nirutha Chetan Kumar, George D’Souza, Marijke P. Baltissen, Joost H. A. Martens, Jorge Domínguez-Andrés, Yang Li, Annapurna Vyakarnam, Mihai G. Netea

**Affiliations:** a Department of Internal Medicine, Radboud Center for Infectious Diseases, Radboud University Medical Centre, Nijmegen, The Netherlands; b Radboud Institute for Molecular Life Sciences, Radboud University Medical Center, Nijmegen, The Netherlands; c Centre for Individualised Infection Medicine (CiiM), a joint venture between the Helmholtz Centre for Infection Research (HZI) and Hannover Medical School (MHH), Hannover, Germany; d TWINCORE Centre for Experimental and Clinical Infection Research, a joint venture between the Helmholtz Centre for Infection Research (HZI) and the Hannover Medical School (MHH), Hannover, Germany; e Department of Molecular Biology, Radboud University, Radboud Institute for Molecular Life Sciences, Nijmegen, The Netherlands; f Laboratory of Immunology of HIV-TB Co-infection, Centre for Infectious Disease Research, Indian Institute of Science, Bangalore, India; g Peter Gorer Department of Immunobiology, School of Immunology and Microbial Sciences, Faculty of Life Sciences & Medicine, Guy’s Hospital, King’s College London, London, United Kingdom; h Department of Immunology and Metabolism, Life & Medical Sciences Institute, University of Bonn, Bonn, Germany; University of Minho

**Keywords:** COVID-19, immune response, viral susceptibility, BCG vaccination

## Abstract

During the coronavirus disease 2019 (COVID-19) pandemic, large differences in susceptibility and mortality due to severe acute respiratory syndrome coronavirus 2 (SARS-CoV-2) infection have been reported between populations in Europe and South Asia. While both host and environmental factors (including Mycobacterium bovis BCG vaccination) have been proposed to explain this, the potential biological substrate of these differences is unknown. We purified peripheral blood mononuclear cells from individuals living in India and the Netherlands at baseline and 10 to 12 weeks after BCG vaccination. We compared chromatin accessibility between the two populations at baseline, as well as gene transcription profiles and cytokine production capacities upon stimulation. The chromatin accessibility of genes important for adaptive immunity was higher in the Indians than in the Europeans, while the latter had more accessible chromatin regions in genes of the innate immune system. At the transcriptional level, we observed that the Indian volunteers displayed a more tolerant immune response to stimulation, in contrast to a more exaggerated response in the Europeans. BCG vaccination strengthened the tolerance program in the Indians but not in the Europeans. These differences may partly explain the different impact of COVID-19 on the two populations.

**IMPORTANCE** In this study, we assessed the differences in immune responses in individuals from India and Europe. This aspect is of great relevance, because of the described differences in morbidity and mortality between India and Europe during the pandemic. We found a significant difference in chromatin accessibility in immune cells from the two populations, followed by a more balanced and effective response in individuals from India. These exciting findings represent a very important piece of the puzzle for understanding the COVID-19 pandemic at a global level.

## INTRODUCTION

The immune response to pathogens is elicited and regulated through a series of pathways shaped by genetic and environmental factors. While the genetic component is constant, environmental factors continuously change during an individual’s life course, and their cumulative effect regulates the immune response (1). These environmental factors include lifestyle, diet, vaccinations, infections, and treatments. By extension, this leads to a great variation in response to pathogens at the individual level. Subsequently, such heterogeneity at the individual level translates into distinct responses in various populations, leading to differences in disease outcome (2, 3).

During the coronavirus disease 2019 (COVID-19) pandemic, striking observations were made regarding differences in the impact and outcome of severe acute respiratory syndrome coronavirus 2 (SARS-CoV-2) infection between populations. It has been reported that while developing countries such as India and countries in sub-Saharan Africa had a high number of COVID-19-related deaths, the case/fatality ratio was lower than that in populations in the developed nations of Western Europe and North America (4) (https://coronavirus.jhu.edu/map.html), despite possible biases due to underreporting. Several mechanisms have been suggested to play a role, such as cross-reactive humoral and cellular lymphocyte responses to other coronaviruses or virus-virus interactions; one study examined the serological cross-reactivity of COVID-19-naive people from sub-Saharan Africa against SARS-CoV-2 and found a high prevalence of preexisting serological cross-reactivity to the novel virus (5). Another hypothesis proposed an increased level of natural resistance to explain, at least partly, the interpopulational variation in response to SARS-CoV-2, resistance that can be induced by local infectious pressure (6). Little is known, however, about the potential differential regulation of immune responses to SARS-CoV-2 or other viruses in various populations.

Considering these observations relevant for the COVID-19 pandemic, we aimed to understand the differences in the antiviral immune responses of Indian and European individuals to the SARS-CoV-2 and influenza viruses. In addition, we studied whether these responses are affected by Mycobacterium bovis BCG vaccination, which has been recently suggested to increase host defense against heterologous infections such as COVID-19.

## RESULTS

### Chromatin accessibility profiles differ in Indian and European individuals.

**FIG 1:**
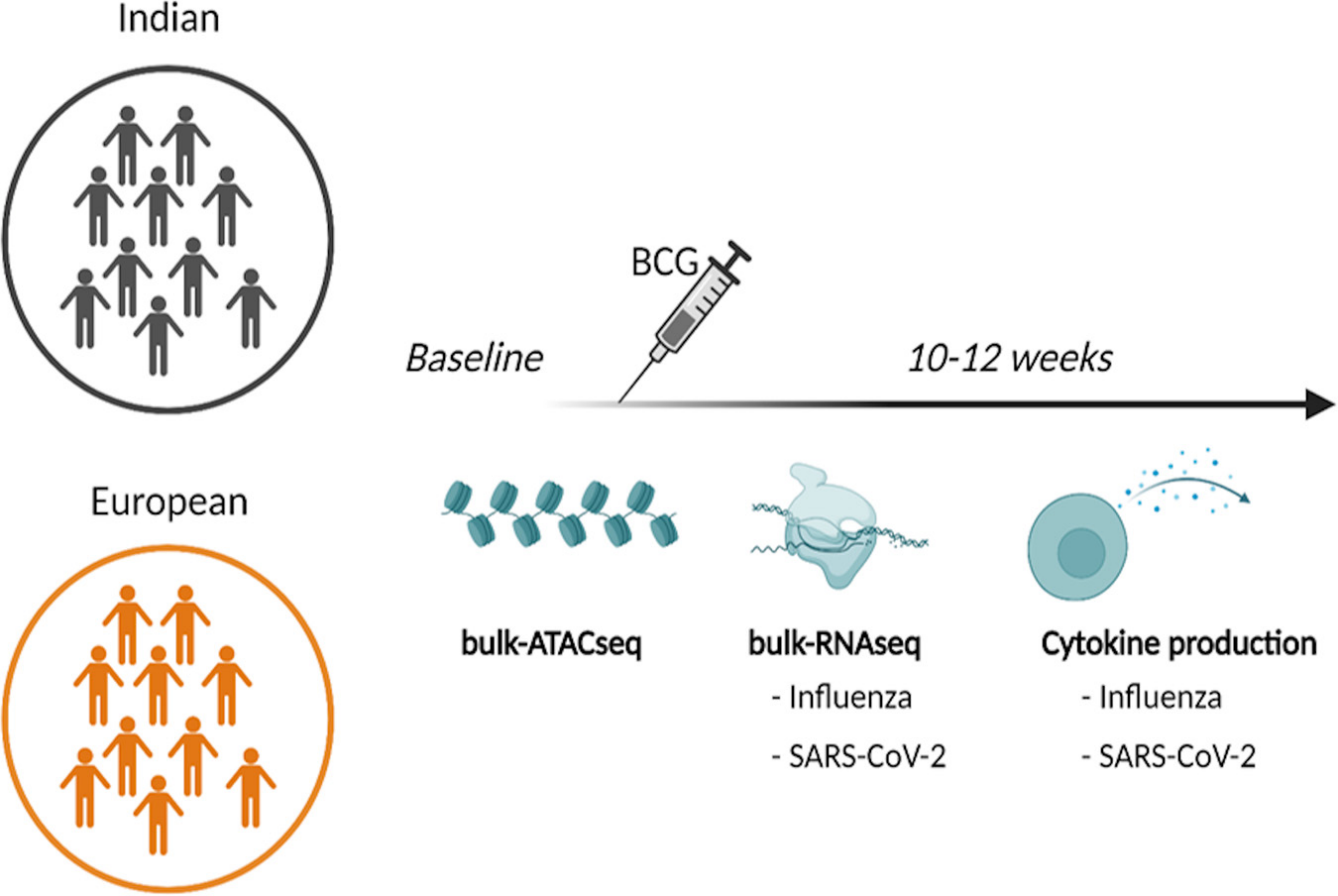
Study design schematic. RNAseq, transcriptome sequencing.

We obtained peripheral blood mononuclear cells (PBMCs) from 10 Indian (6 men, 4 women; all between 20 and 30 years of age) and 10 European (5 men, 5 women; all also between 20 and 30 years of age) individuals before and 10 to 12 weeks after BCG vaccination (Fig. 1). While the European individuals were BCG vaccine-naive before the vaccination given during the study, the Indian individuals were known to have been given the BCG vaccine at birth as a part of their national vaccination program.

**FIG 2:**
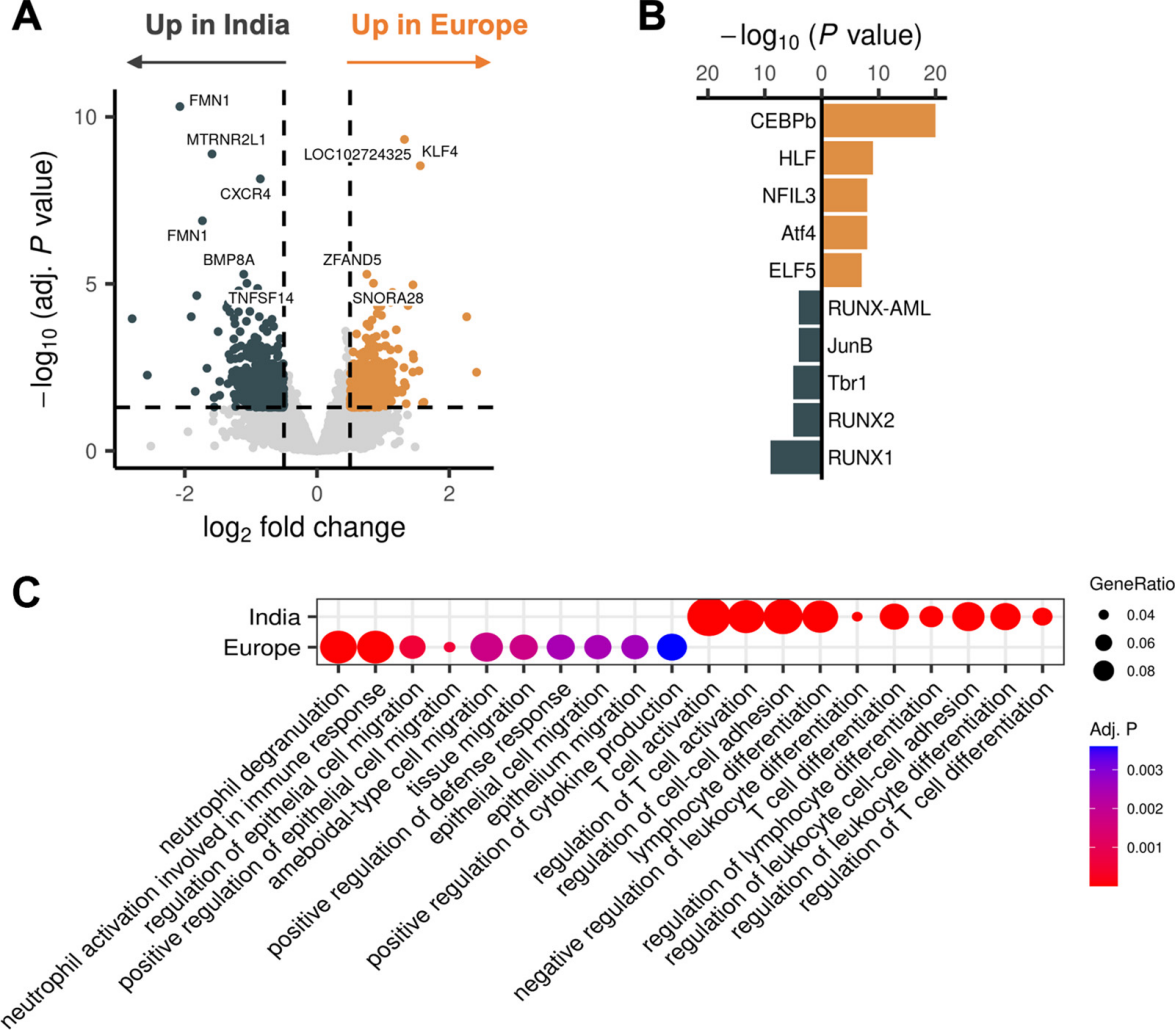
Chromatin accessibility profiles in Indian and European individuals, demonstrating activity in different compartments of the immune system. (A) Volcano plot showing the difference in chromatin accessibility per peak between the European and Indian individuals at baseline (absolute log_2_-transformed fold change, >0.5; adjusted [adj.] *P* value, <0.05). The most significant peaks are labeled with their closest gene based on HOMER annotation. The golden peaks are upregulated in the European individuals, while the dark gray circles are upregulated in the Indian individuals. (B) Bar plot of the transcription factor motif enrichment among the differentially accessible peaks. (C) Dot plot of the gene ontology (GO) term enrichment for genes linked to differentially accessible peaks.

First, we investigated the differences in chromatin accessibility in the unstimulated PBMCs from the Indian and European individuals before BCG vaccination (during homeostasis). Differential peak analysis showed substantial differences between the two populations (Fig. 2A).

Subsequently, motif enrichment analysis of the differentially accessible peaks in each population identified several overrepresented transcription factor-binding motifs unique to each population (Fig. 2B). In the European population, we found the transcription factors CCAAT enhancer binding protein beta (CEBPB) and hepatic leukemia factor (HLF) to be overrepresented. CEBPB regulates the expression of genes that are involved in immune pathways and inflammatory responses (7, 8). In the Indian population, multiple transcription factors of the RUNX family, which are critical mediators of hematopoiesis, were significantly enriched (9). Furthermore, the transcription factor JunB is involved in a wide range of immunological processes, including macrophage activation (10) and regulatory T-cell homeostasis (11).

Using gene set enrichment analysis of genes in close proximity to differentially accessible peaks (Fig. 2C), we observed striking differences between the enriched pathways in each population. The open peaks in the European population were more enriched in genes important for innate immune-related processes, whereas the open peaks in the Indian population were highly related to adaptive immunity and T-cell immunity.

### Antiviral response is more regulated in Indian individuals than in European individuals.

**FIG 3:**
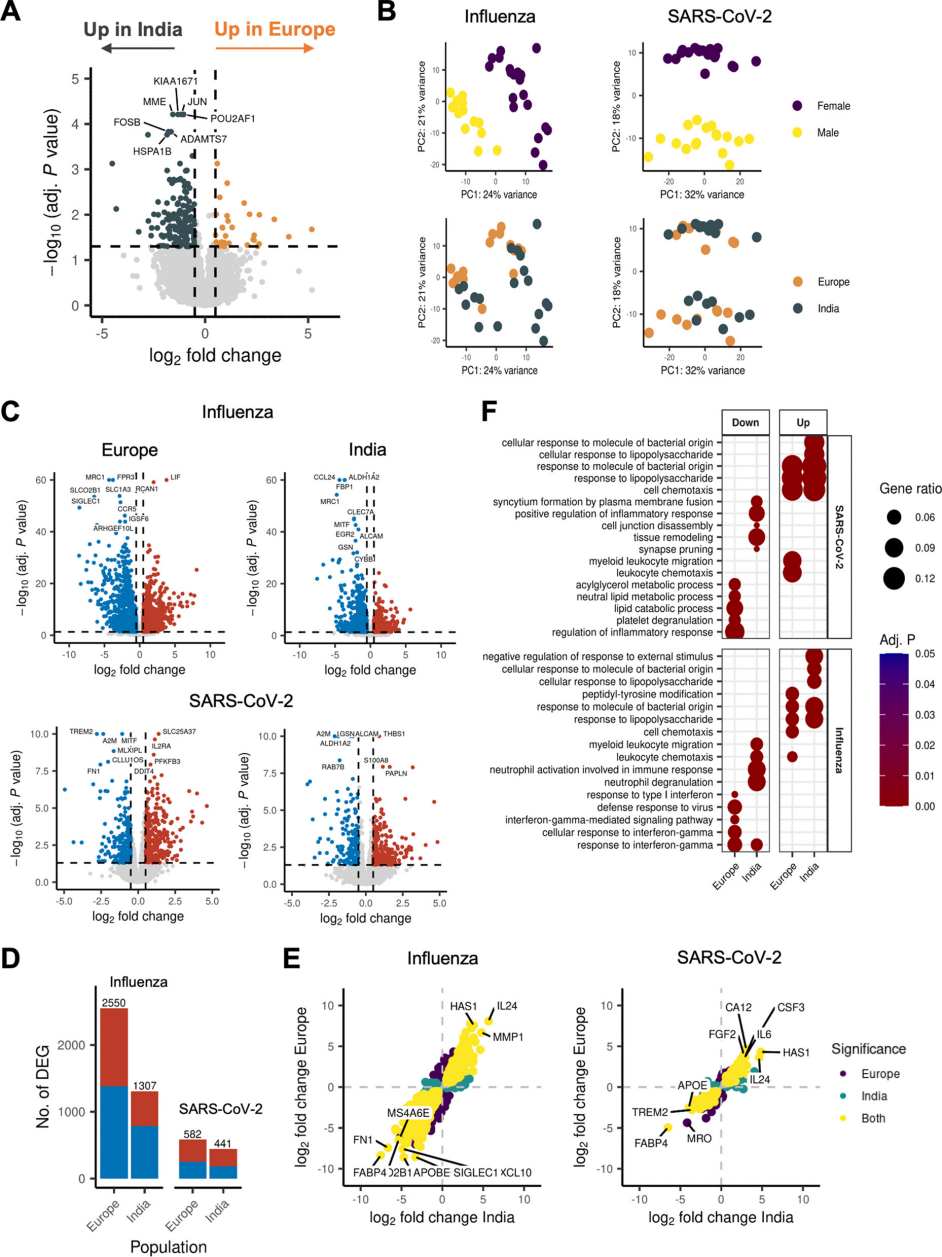
Transcriptional response to SARS-CoV-2 and influenza virus differs in magnitude between Europeans and Indians. (A) Volcano plot of the baseline differences between the European and Indian individuals (absolute log_2_-transformed fold change, >0.5; adj. *P* value, <0.05). (B) Principal-component analysis (PCA) of the response to stimulation. Each point represents a sample. Samples included are those that were influenza/SARS-CoV-2 stimulated. (C) Volcano plots showing the response to influenza and SARS-CoV-2 stimulation compared to RPMI-treated PBMCs. (D) Bar plot of the number of differentially expressed genes per stimulation and population. Blue indicates downregulation; red indicates upregulation. (E) Consistency of the response to viral stimulation between the European and Indian populations. Each point represents a gene, plotted by the log_2_ fold change per stimulation, compared to RPMI-treated PBMCs in the Indians and Europeans. (F) Dot plot of GO term enrichment results of the differentially expressed genes per stimulation and population.

We explored the transcriptional differences between the Europeans and Indians under untreated conditions and upon stimulation with heat-inactivated SARS-CoV-2 and influenza, respectively. Under untreated conditions, we found substantial differences between the populations at the transcriptional level (Fig. 3A). Among the most significantly differentially expressed genes (DEGs) were FOSB and JUN, consistent with enrichment of the JunB transcription factor from our aforementioned analysis.

For PBMCs stimulated with either SARS-CoV-2 or influenza, principal-component analysis (PCA)-based dimensionality reduction revealed that the individual’s sex, and not their geographical origin, drove more strongly the variance between samples (Fig. 3B). Using linear models corrected for age and sex, we estimated the viral response and found that overall, both viruses led to a considerable response at the transcriptional level (Fig. 3C). Investigating the viral response in both populations revealed that the Europeans were more responsive to viral stimuli than were the Indians, evidenced by a higher number of differentially expressed genes (Fig. 3D) and a larger magnitude of up- and downregulation (Fig. 3E). These effects were more visible for the influenza virus than for SARS-CoV-2. Finally, we asked which transcriptional modules were activated in response to viral stimulation and how these differed between the populations. Figure 3F shows both the shared and population-specific transcriptional modules significantly enriched upon stimulation. In the Europeans, more pathways related to type 1 interferons and interferon gamma (IFN-γ) signaling were downregulated in response to stimulation, while in the Indians, pathways related to myeloid cell migration and neutrophil activation, both important features of inflammation, were downregulated more strongly.

**FIG 4:**
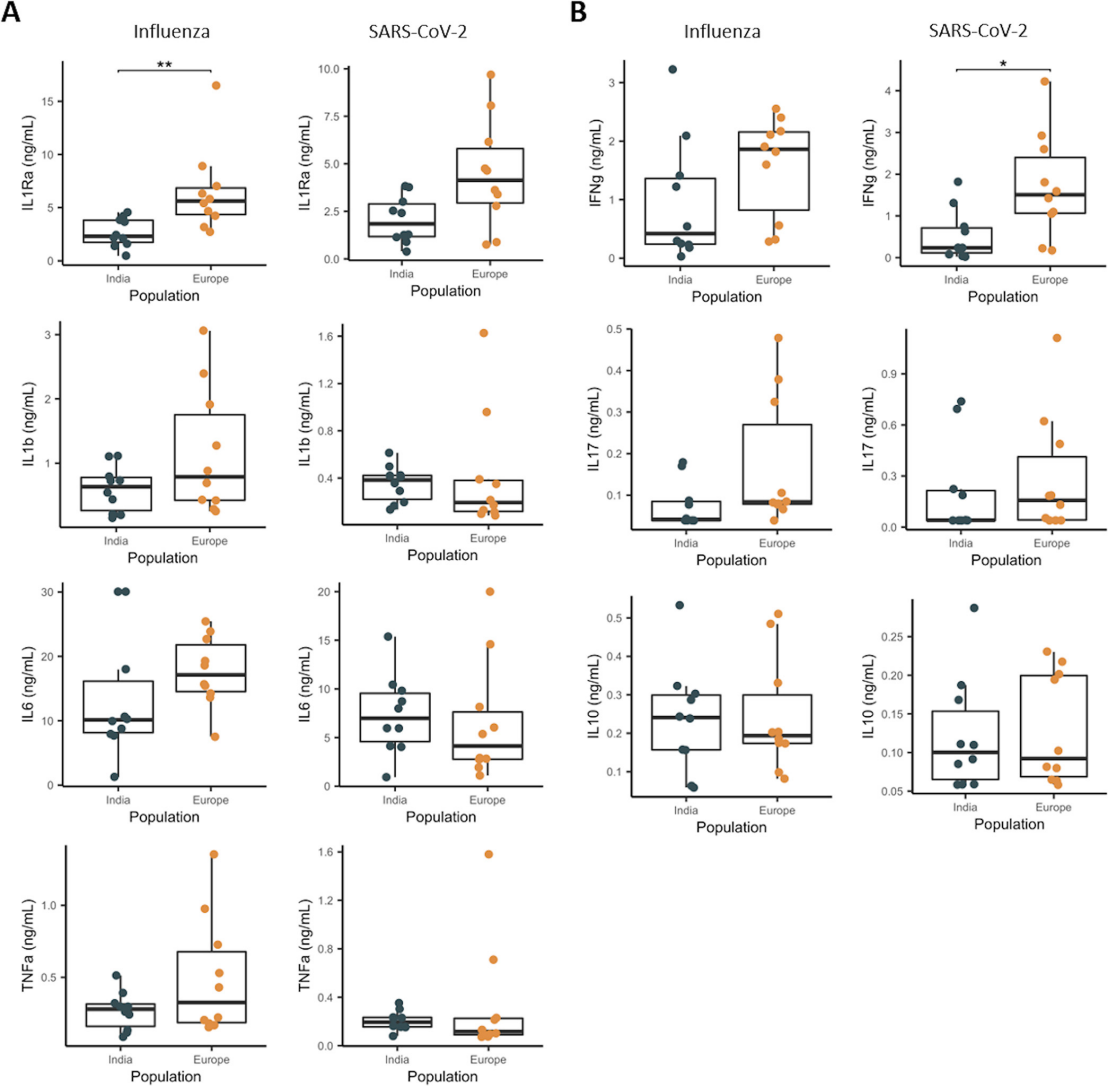
Cytokine production in Indian and European individuals. (A) Proinflammatory cytokine production following 24 h of stimulation, measured by ELISA (Mann-Whitney U test; *n* = 10). (B) T-cell cytokine production following 7 days of stimulation, measured by ELISA (Mann-Whitney U test; *n* = 10).

In agreement with the transcriptional data, cytokine production overall was higher in the European individuals than in the Indian group. Pro- and anti-inflammatory cytokines (interleukin 1Ra [IL-1Ra], IL-1b, IL-6, tumor necrosis factor alpha [TNF-α]) were all more strongly elevated in the European group after 24 h of stimulation with the influenza virus. This difference reached significance (two-sided Wilcoxon rank-sum test; *P* = 0.001) in the IL-1Ra readout, despite the small sample size. In contrast, in response to SARS-CoV-2, the production of these cytokines showed wide variation. There was a clear difference in IL-1Ra production, as the Indian individuals had lower production than the European individuals. However, production of the proinflammatory cytokines IL-1b, IL-6, and TNF-α was higher in the Indian individuals in response to SARS-CoV-2 (Fig. 4A). Nevertheless, these differences were not statistically significant. The tendency of cells isolated from the European volunteers to produce more proinflammatory cytokines may partially derive from their higher monocyte percentage compared to the Indian individuals (see Fig. S1 in the supplemental material). Next, we checked T-cell-derived cytokines to understand the antigen-specific responses better (Fig. 4B). Production of the antiviral cytokine IFN-γ was higher in the European group, but statistical differences were reached only in response to SARS-CoV-2 stimulation. This pattern remained the same for IL-17: lower production in the Indian individuals and higher in the Europeans, but the difference was not significant. There was no difference in IL-10 production between the two populations. These data show that the Indian individuals had an overall lower responsiveness to viral stimuli, i.e., influenza and SARS-CoV-2.

### Long-term effect of BCG vaccination on the response to influenza and SARS-CoV-2 stimulation of PBMCs from the Indian and European volunteers.

As a live-attenuated vaccine, BCG is known to induce long-term effects on the innate immune system and provide nonspecific protection against various infections (12). This phenomenon has been termed “trained immunity” and is functionally characterized by augmented responsiveness of innate immune cells (13). Given that the Indian and European individuals had significantly different chromatin accessibilities and differential transcriptional and functional responses to viral stimulation, we hypothesized that they would also have distinct responses to BCG vaccination. Therefore, we analyzed the viral response of the PBMCs of the Indian and European individuals before and 10 to 12 weeks after BCG vaccination (Fig. S2).

**FIG 5:**
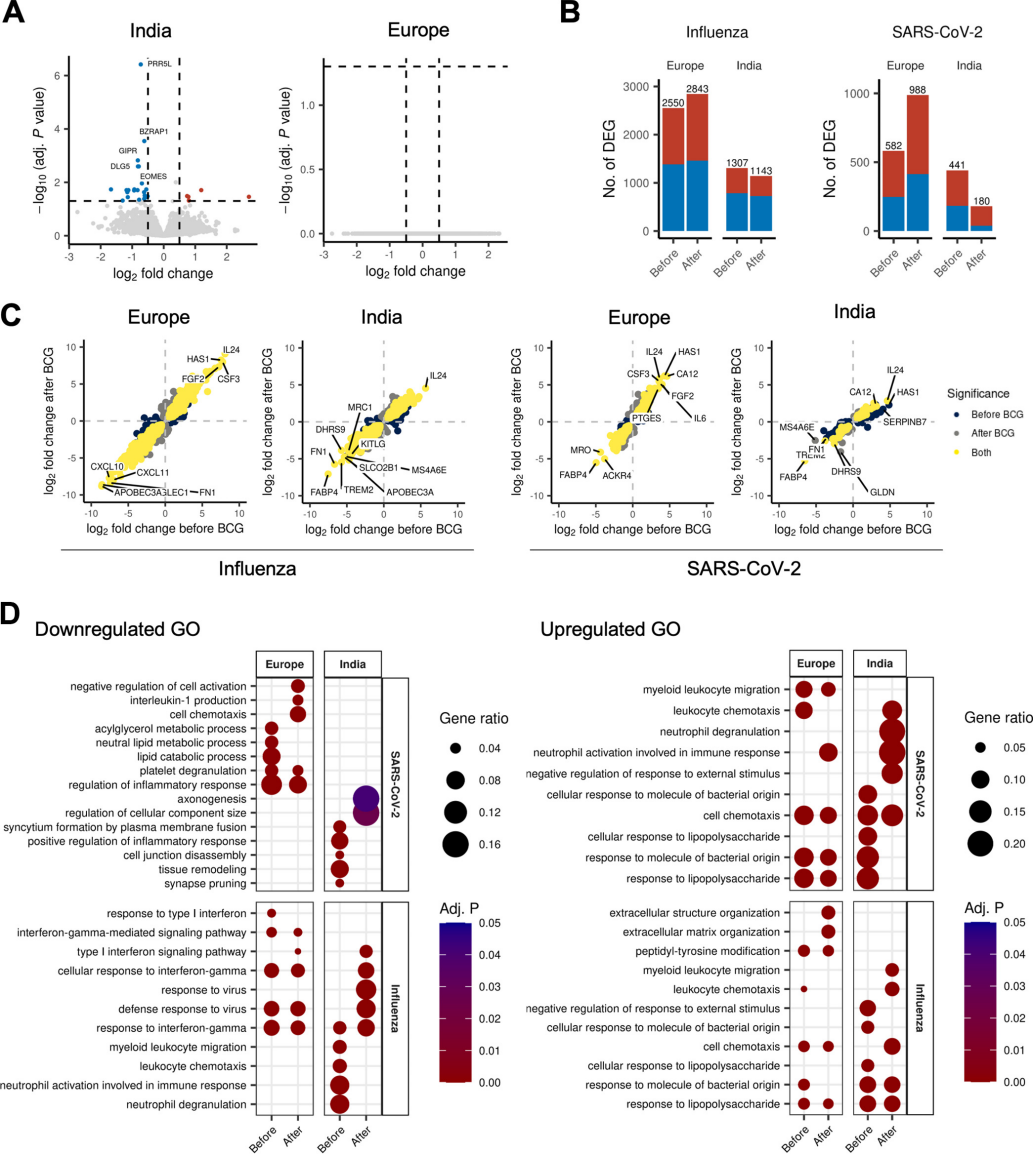
Transcriptional response to viral stimulation before and after BCG vaccination. (A) Volcano plot showing the effect of BCG vaccination on the transcriptome (absolute log_2_-transformed fold change, >0.5). (B) Bar plot indicating the number of differentially expressed genes for each viral stimulation compared to RPMI-treated PBMCs before and after BCG vaccination. Blue indicates downregulation; red indicates upregulation. (C) Consistency of the response to viral stimulation before and after BCG vaccination. Each point represents a gene, plotted by the log_2_ fold change per stimulation before and after BCG vaccination. (D) Dot plot of GO term enrichment results showing the transcriptional modules that are regulated following stimulation, before and after BCG vaccination.

Transcriptional analysis of the unstimulated PBMCs before and after BCG vaccination showed minimal differences in the Indian group, whereas no differences were observed in the European group (Fig. 5A). On the other hand, viral stimulation induced significant changes in the transcriptome before and after BCG vaccination. The magnitude of response to either influenza or SARS-CoV-2 was overall greater in the European group than in the Indian group, both before and after BCG vaccination (Fig. 5B). The number of DEGs after influenza virus stimulation changed significantly (from 1,307 to 1,143) in the Indian individuals compared to the Europeans (from 2,550 to 2,843; Fisher’s exact test, *P* < 0.00001).

The response to SARS-CoV-2 showed an even clearer difference. In the European group, the number of DEGs increased substantially after BCG vaccination (from 582 to 988). Surprisingly, the number of DEGs strongly decreased in Indian individuals after BCG vaccination (from 441 to 180) (Fig. 5B). We performed a systemic comparison of the direction of regulation per gene, and our analysis revealed that most genes were regulated in a consistent fashion between the groups, although population-specific genes were also observed. This pattern was consistent for both stimulations (Fig. 5C).

Gene ontology (GO) enrichment analysis of the differentially expressed genes upon stimulation underlined the differences in response to viral stimuli between the European and Indian individuals (Fig. 5D). These results align with the previous data showing that the European and Indian individuals had different chromatin accessibility profiles related to innate and adaptive immunity, respectively. Stimulation with SARS-CoV-2 in the European group downregulated metabolic processes such as lipid metabolism and regulation of inflammatory response before BCG vaccination. However, this shifted toward inhibition of the IL-1 production pathway and negative regulation of cell activation with the persistent presence of regulation of inflammatory response after BCG vaccination. In contrast, the Indian individuals had no overlap with the active pathways in the European individuals. However, positive regulation of the inflammatory response was downregulated by SARS-CoV-2 stimulation in cells collected before BCG vaccination from the Indian individuals but disappeared after BCG vaccination. Interestingly, the Indian volunteers showed the most significant downregulation in response to SARS-CoV-2 following BCG vaccination, in regulation of cellular component size and axonogenesis processes (Fig. 5D). There was an emphasis on the induction of myeloid leukocyte migration and leukocyte chemotaxis in the European individuals before BCG vaccination, which was absent in the Indian individuals. Interestingly, the Indian volunteers had a very distinct response to SARS-CoV-2 after BCG vaccination. While the vaccination boosted the expression of genes important for neutrophil function in the Indian individuals, it did not change the balanced distribution of different immunological response pathways in the European individuals.

Furthermore, the pathways related to IFN-γ that were downregulated in response to influenza did not differ in the European individuals before and after BCG vaccination. On the other hand, in the case of the Indian individuals, neutrophil degranulation and the IFN-γ pathway response were downregulated before BCG vaccination, while the defense response to virus-associated pathways was downregulated after the vaccination. When we checked the upregulated pathways, there was no specific significant pattern for the European individuals before and after BCG vaccination.

**FIG 6:**
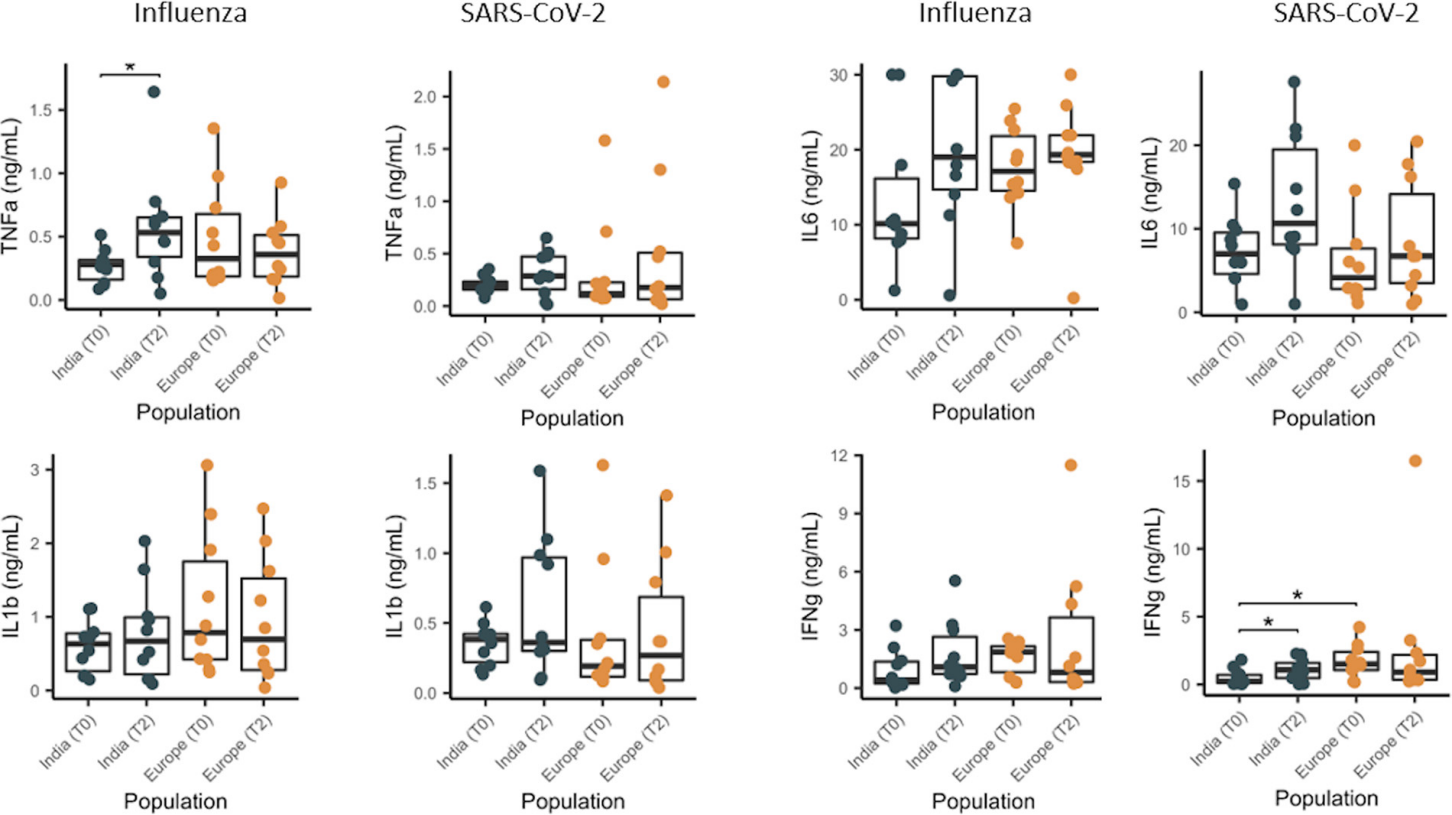
Indian and European individuals display distinctive differences in cytokine production before and after BCG vaccination. Cytokine production increased more prominently in the Indian individuals than in the European individuals after BCG vaccination. T0, baseline; T2, 10 to 12 weeks postvaccination; *n* = 10. The Wilcoxon paired test was used for comparison within groups; the Mann-Whitney U test was used for comparison between groups.

After analyzing the effect of BCG vaccination on transcriptional profiles of both groups of individuals, we sought to understand the functional outcome of the process. We measured proinflammatory cytokines (IL-6, TNF-α, IL-1β) and IFN-γ from stimulated PBMCs of both populations before and after BCG vaccination (Fig. 6). As we presented above in the transcriptional data with higher innate immune activation in the Indian group after BCG vaccination, proinflammatory cytokine secretion was elevated in these individuals after vaccination in response to influenza and SARS-CoV-2. On the other hand, in the European individuals, we were not able to identify significant changes in the innate immune responses after vaccination, most likely due to the large variation. This pattern was similar for IFN-γ production but with a significant increase in the Indian group following vaccination in response to SARS-CoV-2.

## DISCUSSION

In the present study, we explored the differences in antiviral immune responses between individuals of European and Indian origin before and after BCG vaccination. Our analyses showed divergent viral responses between the populations. Identical transcriptional modules were activated upon stimulation, yet the extent of activation was greater in the Europeans, while a more regulated tolerant response was observed in the Indians. Vaccination with BCG, which has been proposed to induce heterologous protective antiviral responses (12), also induced different effects in the two populations; interestingly, the overall transcriptional responses were larger in cells from the European donors after *ex vivo* stimulation with heat-inactivated SARS-CoV-2 or influenza viruses, while becoming more regulated and limited in the Indians. This resulted in a more focused and likely more effective functional immune response against viruses in individuals of Indian origin.

There are several hypotheses for the different impact of COVID-19 observed in the Indian and European populations. First, Indians likely experience more intense infectious pressure compared to Europeans. This may lead to a more activated state of the innate immune response during homeostasis, which potentially “primes” the innate immune cells to respond more adequately to stimulation, a process termed “trained immunity” (13). Furthermore, constant infectious pressure may lead to a more developed adaptive immune system and consequently, a more controlled immune response to viral challenges. Together, the cumulative effect of all past vaccinations, infections, and other viral challenges substantially influence the present human immune response (14). Second, substantial differences exist in the genetic background between the two populations. While we did not consider the genetic aspect in the current study, previous work set out to map the genetic influence of viral infection (15) and cytokine production (16) between genetically divergent populations. In a recent study comparing the antiviral immune responses of individuals of European and African ancestry, the individuals with a high percentage of European ancestry had an immune response associated with an increased type I interferon response upon influenza stimulation, compared to the African population (15). This could be taken as confirmation of our results, although the comparison was between different populations. Third, differences in cross-reactive T-cell populations may provide a degree of adaptive immunity to influenza or SARS-CoV-2. Indeed, studies have shown that sub-Saharan African populations had higher serological cross-reactivity to SARS-CoV-2, even before the start of the pandemic (5). Altogether, both host and environmental factors may determine the differences between the basal chromatin accessibility and antiviral responses in Europeans and Indians, and future studies are warranted to investigate this.

BCG vaccination has been shown to induce beneficial long-term effects on the innate arm of the immune system and provide nonspecific protection against various infections (12). A recent large phase 3 BCG trial in elderly Europeans failed to show a significant difference in the total number of infections between vaccinated and nonvaccinated individuals (17), although a positive effect on mortality has been suggested (18). In contrast, initial information suggests positive effects of BCG vaccination in a phase 3 clinical trial of elderly individuals in India (19, 20). Our data suggest that such positive effects may be related to a more tolerant, yet effective, anti-SARS-CoV-2 immune response in the volunteers from the Indian population after vaccination with BCG, which would avoid hyperinflammation and subsequent severe forms of the disease. On the other hand, excessive increase of cytokine production can also be pathogenic and lead to a wide range of pathological conditions.

There are several limitations to be considered. First, all Indian individuals receive the BCG vaccination at birth, but it had not been administered to the Dutch participants. Therefore, the current study shows the effect of BCG revaccination in Indians, rather than their first exposure to the vaccine. Second, we cannot exclude minor differences in the collection and isolation procedures in India and the Netherlands. However, we tried to minimize these differences by performing the stimulation experiments and sequencing at the same time, in the same laboratory, by the same researchers. In addition, one aspect that was different between the two arms of the study is the use of different BCG strains in the Netherlands (BCG-Denmark) and India (BCG-Russia), and this may account for some of the differences. Third, despite the clear and statistically significant differences we observed here, the current study was limited by the number of participants. We did not have access to more biological samples from volunteers in India due to logistical aspects related to the pandemic itself. Later during the pandemic, when accessibility improves, vaccination programs with specific anti-COVID-19 vaccines should be taken into account. Further studies, especially those investigating the genetic aspect of differential antiviral responses between populations, should focus on larger population-based cohorts from different genetic ancestries to properly power their studies.

In summary, this study shows a significantly different antiviral response between Indian and European individuals. The individuals of Indian ancestry displayed a less exuberant transcriptional response upon exposure of their immune cells to viral stimulation compared to the Europeans. Furthermore, BCG revaccination further promoted a more tolerant but effective immune response to SARS-CoV-2 in the Indian, but not European, individuals. These differences could be driven by infectious burden, previous vaccinations, lifestyle, heterologous leukocyte immune cell populations, trained immunity, etc., and future studies are warranted to explore these possibilities in greater detail.

## MATERIALS AND METHODS

### Study design and sample collection.

The Indian participants were healthy health care workers at St. John’s Medical College Hospital (Bangalore, India), invited to participate between October 2019 and June 2021 (21). All included Indian participants had received the BCG vaccination at birth. All Indian participants tested negative against both the SARS-CoV-2 nucleocapsid (N) protein and the receptor-binding domain (RBD) (see Fig. S3 in the supplemental material). The European participants were healthy, nonsmoking young volunteers undergoing BCG vaccination in the BCG-Booster study. The participants did not have a history of past BCG vaccination. Exclusion criteria were acute illness less than 2 weeks before receiving the BCG vaccine and using medication, including nonsteroidal anti-inflammatory drugs (NSAIDs) but excluding oral contraceptives, less than 4 weeks before vaccination. The female participants were screened for pregnancy. The European participants were enrolled before the beginning of the COVID-19 pandemic and therefore were not screened for SARS-CoV-2 serological status.

### Ethics approval.

This study was performed in accordance with the relevant guidelines and regulations stated in the Declaration of Helsinki. Ethical approval for the Indian arm of the study was given by the Institutional Ethics Committee (IEC) of St. John Medical College Hospital, Bangalore (IEC reference number IEC/1/896/2018). Ethical approval for the European arm of the study was given by the Arnhem-Nijmegen Ethical Committee (number NL58219.091.16).

### PBMC isolation.

Venous blood of the European study participants was collected into 10-mL EDTA tubes (BD Vacutainer, USA). PBMC isolation was performed using density centrifugation of blood diluted 1:1 in phosphate-buffered saline (PBS) and layered over Ficoll-Paque Plus (Cytiva, USA). Then, the PBMC layer was collected and washed twice in cold PBS. After cell counting, the PBMCs were preserved in bovine calf serum (BCS) containing 10% dimethyl sulfoxide (DMSO) in liquid nitrogen for use in the sequencing and stimulation assays. Blood from the Indian study participants was collected in Na-heparin tubes (BD, Franklin Lakes, NJ, USA) and diluted 1:1 with PBS (Gibco by Life Technologies, Washington, DC, USA) plus 2% fetal bovine serum (FBS) (Gibco). PBMC isolation was performed using 15-mL Accuspin tubes (Sigma-Aldrich) by density centrifugation, following the manufacturer’s instructions. PBMCs from the buffy coat were washed twice with PBS plus 2% FBS, resuspended at 10 × 10^6^ cells/mL in a cryopreservation medium (90% FBS and 10% DMSO), incubated overnight at −80°C (in a Mr. Frosty freezing container; Nalgene, Rochester, NY, USA), and stored in liquid nitrogen until further analysis.

The PBMCs from both the Dutch and Indian participants were thawed and washed twice with 10 mL Dutch modified RPMI 1640 medium (Invitrogen, USA; catalog number 22409031) containing 50 μg/mL gentamicin (Centrafarm, The Netherlands), 1 mM sodium-pyruvate (Thermo Fisher Scientific, USA; catalog number 11360088), and 2 mM GlutaMAX (Thermo Fisher Scientific; catalog number 35050087) supplemented with 10% bovine calf serum (Fisher Scientific, USA; catalog number 11551831). Afterward, the cells were counted using a Sysmex XN-450 device. The cells were divided for ATAC-seq (assay for transposase-accessible chromatin with high-throughput sequencing) preparation (0.5 × 10^5^ cells/sample) and for the stimulation experiments (4 × 10^5^ cells/well).

### Bulk ATAC sequencing.

Untreated PBMCs were subjected to tagmentation to prepare for ATAC-seq. The cells were lysed with TDE1 (tagment DNA enzyme; Illumina; catalog number 20034197). After lysis, the DNA fragments were eluted following the Qiagen MinElute kit protocol. The DNA was subsequently PCR amplified using KAPA HiFi HotStart ready mix (Kapa Biosystems) and Nextera index kit (Illumina) primers, followed by reverse-phase 0.65× solid-phase reversible-immobilization (SPRI) bead purification and QIAquick spin column (Qiagen) purification. The amplified DNA libraries were sequenced using an Illumina NextSeq 500 instrument with a read length of 38 bp.

### PBMC stimulation experiments.

PBMCs (4 × 10^5^ cells/well) were laid in sterile, round-bottom, 96-well tissue culture treated plates (VWR, the Netherlands; catalog number 734-2184) in Dutch modified RPMI 1640 medium containing 50 μg/mL gentamicin, 1 mM sodium-pyruvate, and 2 mM GlutaMAX supplemented with 10% human pooled serum. The cells were rested for an hour in a 37°C incubator supplied with 5% CO_2_ before stimulation. The stimulations were performed with two heat-inactivated viral stimuli: SARS-CoV-2 strain Wuhan Hu-1 (2.8 × 10^3^ 50% tissue culture infective dose [TCID_50_]/mL) and influenza California A (3.6 × 10^3^ TCID_50_/mL). The PBMCs were incubated with the stimulants for 24 h to detect IL-1β, TNF-α, IL-6, and IL-1Ra and for 7 days to detect IFN-γ, IL-17, and IL-10. Supernatants were collected and stored at −20°C.

### Bulk RNA sequencing.

PBMCs were lysed with Qiagen RNeasy lysis buffer (RLT) following 24 h of stimulation and stored at −80°C until isolation. RNA was isolated using the Qiagen RNeasy kit, following the manufacturer’s instructions. A total of 200 ng RNA per sample was used for the preparation of RNA sequencing libraries using the KAPA RNA HyperPrep kit with RiboErase (human/mouse/rat [HMR]) (Kapa Biosystems). In short, oligonucleotide hybridization and rRNA depletion, rRNA depletion cleanup, DNase digestion, DNase digestion cleanup, and RNA elution were performed according to protocol. Fragmentation and priming were performed at 94°C for 6 min 30 s. First-strand synthesis, second-strand synthesis, and A-tailing were performed according to protocol. For adapter ligation, a 7-μM stock was used (NEXTflex DNA barcodes; Bioo Scientific). The first and second postligation cleanups were performed according to protocol. For library amplification, 6 cycles were used. Library amplification cleanup was performed using a 0.8× bead-based cleanup. The library size was determined using the high-sensitivity DNA bioanalyzer (Agilent Technologies), the library concentration was measured using the DeNovix double-stranded DNA (dsDNA) high-sensitivity assay. Sequencing was performed using an Illumina NextSeq 500 instrument; 38-bp paired-end reads were generated.

### Quantification and statistical analysis.

**(i) Cytokine production upon stimulation.** The secreted cytokine levels from the supernatants following stimulation were quantified by enzyme-linked immunosorbent assay (ELISA) (IL-1β, catalog number DLB50; TNF-α, catalog number STA00D; IL-6, catalog number D6050; IL-1Ra, catalog number DRA00B; IFN-γ, catalog number DY285B; IL-10, catalog number DY21YB; IL-17, catalog number DY317; R&D Systems, USA), following the instructions of the manufacturer. The Mann-Whitney U test was used to compare cytokine levels between populations and the Wilcoxon paired test for comparison within populations.

**(ii) Bulk ATAC sequencing.** ATAC sequencing reads were preprocessed using the nfcore/atacseq pipeline v1.2.1 (22) implemented in Nextflow v21.04.3 (23) using default settings and the human genome (GRCh38). We considered the consensus peak set called by MACS2 in broad mode. Peaks located on sex chromosomes were removed prior to further analysis, and the remaining peaks were annotated using HOMER (24). Differential peak abundance to compare the populations was performed with linear models using DESeq2 v1.30.1 (25), incorporating each individual’s age and sex in the model. The resulting *P* values were corrected across all peaks using the Benjamini-Hochberg method, and adjusted *P* values of <0.05 were considered significant.

**(iii) Bulk RNA sequencing.** The bulk RNA sequencing reads were preprocessed using the publicly available nfcore/rnaseq v2.0 pipeline (22) with default settings and the human genome (GRCh38). Differential gene expression was implemented with linear models using DESeq2 v1.30.1 (25). The models included the individual’s sex to estimate the BCG vaccination effect and baseline differences between the populations. To compare each viral stimulation against RPMI-treated control samples, a paired design was used that included each individual’s study ID. Genes with total read counts of <20 were removed prior to further analysis. The resulting *P* values were corrected across all genes using the Benjamini-Hochberg method, and adjusted *P* values of <0.05 were considered significant.

### Data availability.

Further information and requests for resources and reagents should be directed to the corresponding author. The bulk transcriptomics and bulk ATAC are freely available and have been deposited at the European Genome-Phenome Archive (EGA), which is hosted by the EBI and CRG, under accession number EGAS00001006417. The codes used in this study are freely available at GitHub (https://github.com/CiiM-Bioinformatics-group/INDIA.git).
