# Supplementary material for: Differences in Immune Responses in Individuals of Indian and European Origin: Relevance for the COVID-19 Pandemic
Source: Microbiol Spectr. 2023 Feb 13;11(2):e00231-23. doi: 10.1128/spectrum.00231-23 (PMC10100912; doi:10.1128/spectrum.00231-23)
Supplement: Supplemental file 1 — Fig. S1 to S3. Download spectrum.00231-23-s0001.pdf, PDF file, 0.1 MB [file spectrum.00231-23-s0001.pdf]

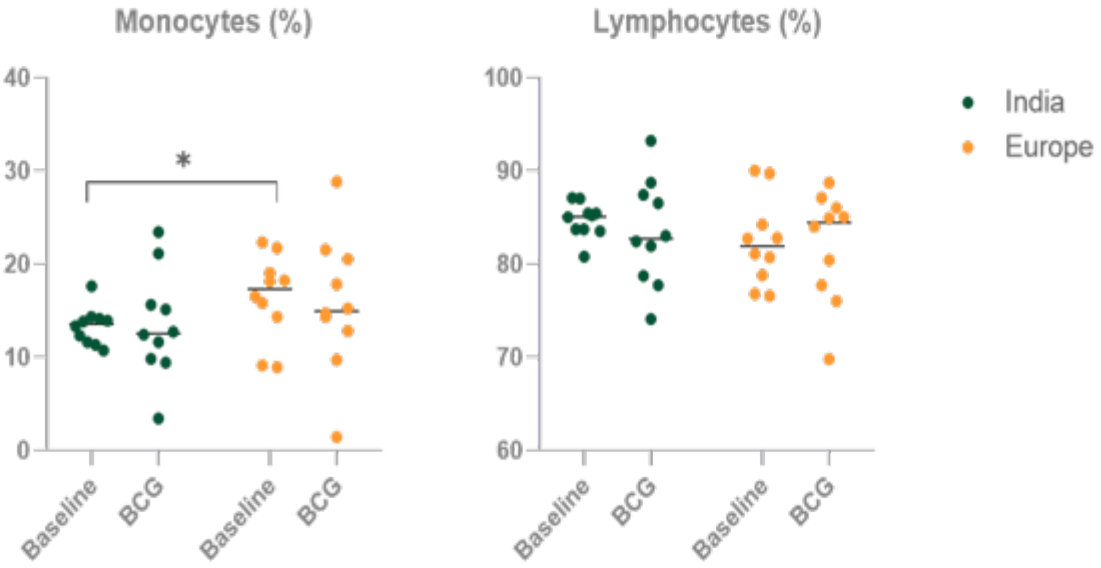

Supplementary Figure 1. Percentage of monocytes and lymphocytes in PBMCs

Proportions of monocytes and lymphocytes in PBMCs counted in before (baseline) and after BCG vaccination samples in the European and Indian cohorts.

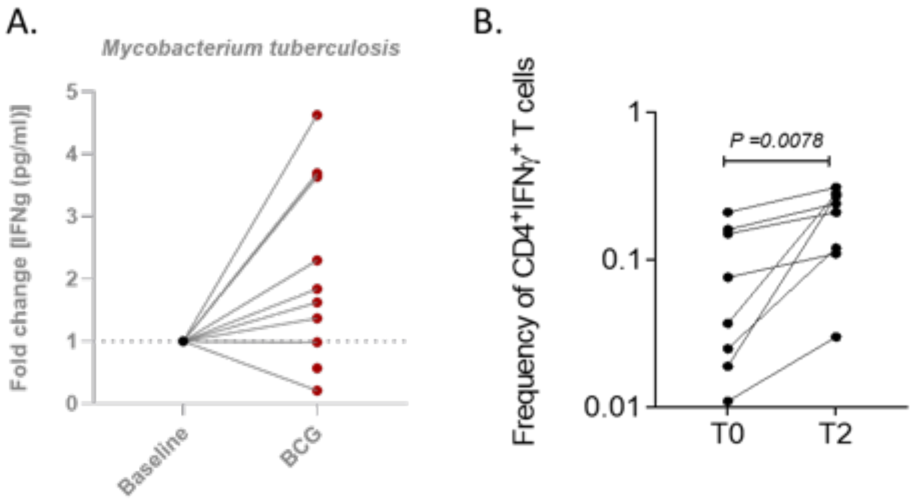

Supplementary Figure 2. BCG vaccine efficacy

A) IFN-gamma production upon ex-vivo stimulation with Mtb, measured before (Baseline) and after BCG vaccination (BCG) in the European cohort. **B)** Frequency of IFN-γ+ CD4+ T cells before (T0) and after (T2) BCG vaccination in the Indian cohort. Wilcoxon matched-pairs signed rank test was used to determine the p-value.

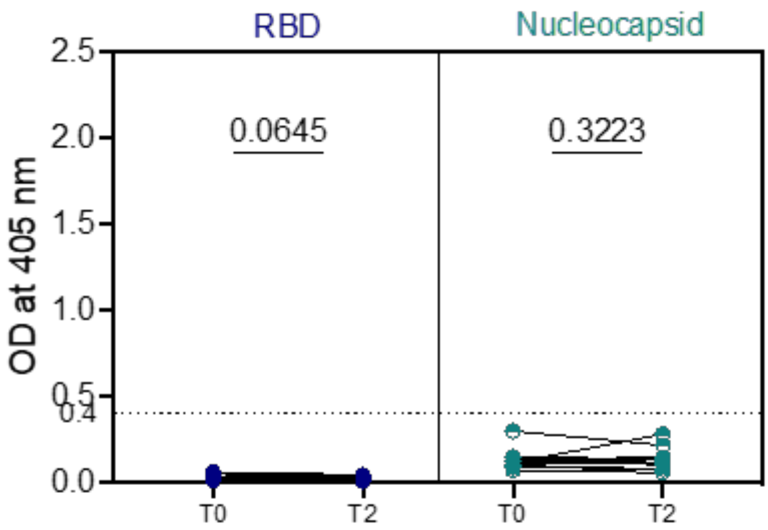

N	10	10	10	10
Median (Range)	0.02520 (0.014-0.063)	0.01845 (0.010-0.047)	0.1198 (0.073-0.301)	0.1155 (0.061-0.283)

Supplementary Figure 3. Serological status of Indian study participants against SARS-CoV-2

India samples are negative for SARS-CoV-2 Spike (RBD) and Nucleocapsid protein-specific IgG. Plasma from donors at baseline (T0) and T2 (8-10 weeks post BCG revaccination) was used to determine antibody titers against the receptor binding domain (RBD) of the spike protein and nucleocapsid protein to assess COVID-19 infection status using a standardized protocol (Rakshit et al., 2022). Wilcoxon matched-pairs signed rank test was used to determine the p-value.
